# Mathematics's role in the grand challenge of deciphering the molecular basis of life

**DOI:** 10.3389/fmolb.2014.00002

**Published:** 2014-03-27

**Authors:** Patrice Koehl

**Affiliations:** Department of Computer Science and Genome Center, University of California at DavisDavis, CA, USA

**Keywords:** biomolecular dynamics, grand challenges, biomolecular shapes, mathematics and biomolecules, RNA structure

## Introduction

Biochemistry, the field that studies biomolecules and their relation to life, is a primary beneficiary of the flood of data that is emerging from the new technologies that allow us to observe biological systems at spatial and temporal scales never achieved before. It remains extraordinarily difficult to grasp the implications and to develop experimentally testable predictions from such complex data. Most of the data are fragmented and only pertain to either a static vision of biology, or provide information on dynamics over a limited time scale. However, the dynamics of biomolecular systems occurs over a large range of time scales. Thermal fluctuations for example occur in the femtosecond (fs)-to-picosecond (ps) time scale, while tumbling occurs in the nano second (ns) time range. Global molecular rearrangements such as those observed in allosteric regulation take place in the microsecond (μs) to millisecond (ms) time range while folding, binding, diffusion, motility and translocation befall in the ms-s-min time range. Macromolecular synthesis and supramolecular processes are even slower processes occurring in the seconds-to-hours time frame. Furthermore, even simple observations of biomolecular systems can require quantitative analyses that are beyond the repertoire of traditional methods available to experimentalists. The scarcity of comprehensive methods is even more acute with regard to answering fundamental questions of the chemistry and physics underlying the multitude of phenomena that define biomolecular systems and their interactions. The current views of the relationship between biomolecular structure and function for example remain fragmented. We know of their sequences, more and more about their structures, and we have information on their biological activities, but we have difficulties connecting these dots into a knowledgeable whole. Thus, biochemistry requires new theoretical and computational approaches toward organizing data into quantitative models. Mathematics (broadly defined) is well positioned to play a major role in these efforts, by working collaboratively with bench biologists, chemists and physicists.

Mathematics has always played an important role in biology, including biochemistry. There is indeed a field of research named “mathematical biology” whose aims are to provide quantitative representations, treatments and modeling of biological processes, using a variety of applied mathematical techniques and tools. By describing biomolecular systems in a quantitative manner, their behavior can be better simulated, and hence properties can be predicted that might not be accessible to the experimenter. Mathematical disciplines such as probability theory, statistics, combinatorics, dynamical systems, and the study of differential equation have traditionally provided the tools for such quantitative analyses. The ongoing transformation of biology into a quantitative science is increasing the need, however, for new tools and new theories. As a consequence, I expect that mathematical areas such as calculus, linear algebra, abstract algebra, graph theory, algebraic geometry, topology, and coding theory will play an increasing role in the new era for biology.

To get a better understanding of the challenges that lie ahead of us, it is worth considering a simple example with significant consequences for human health. Dengue virus is a positive sense RNA virus responsible for dengue fever, a tropical infectious disease whose incidence has increased drastically over the last decades. There are currently no prophylactic treatments against dengue fever, with the exception of eliminating the vector mosquitoes. The genome of dengue virus encodes for 10 different proteins. A perhaps surprising idea that has crystallized from years of studies of dengue virus is that its biology is deeply encoded in the dynamics of these proteins. For example, the envelope protein E on the surface of dengue viruses undergoes a change in conformation upon entering the endosome following the infection of a cell. This structural change is ultimately responsible for the release of the virus genome in the cell (Bressanelli et al., 2004; Modis et al., 2004). The surface of dengue virus is also known to be dynamic (Lok et al., 2008). An antibody that locks the virus surface in a static state blocks infection by preventing attachment of dengue virus to cells (Teoh et al., 2012). These are all key indicators that the dynamic movement of dengue surface proteins is important for cell attachment and ultimately its virulence. Our experimental knowledge of this dynamics that occurs at different time scales, however, is limited. How are structure and dynamics connected for the dengue virus? How do we connect dynamics to the biology of viral entry into cells? What is the connection between dynamics and to the inhibition of dengue virus with antibodies? These are key questions that are vital to the design of vaccines as well as to the identification of prophylactic antibodies.

Deciphering the relationships between the structure, function, and dynamics of biomolecules is the grand challenge faced by biochemists in their attempt to unravel the mysteries of life. There are many facets of this challenge that remain unsolved. Of particular interest, the subjects of biomolecular structure prediction and folding remain key challenges for the community of computational and theoretical structural biologists. Interestingly, these areas define a treasure trove of problems where mathematics can prove useful. In the following two sections, I outline some of the mathematical challenges featured in these research areas. Hopefully some of these challenges will be met and discussed in the new Frontiers section, “Mathematics and Biomolecules.”

## Grand challenge I: the mathematics of biomolecular shapes

**Figure 1:**
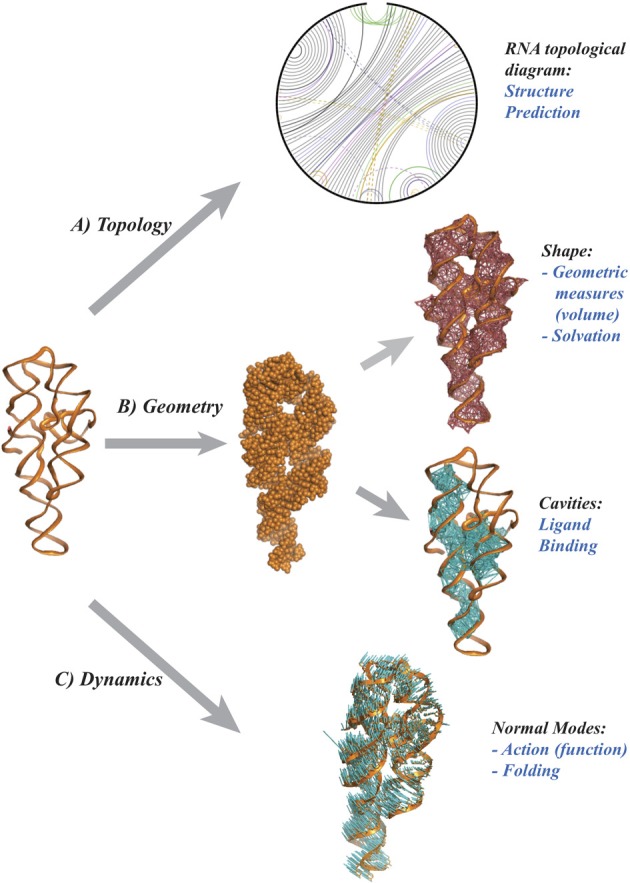
**Topology and Geometry of RNA molecules**. From a pure chemical point of view, a RNA molecule is a polymer of nucleotide residues, The cartoon on the left illustrates the backbone of the P4-P6 group I rybozyme of tetrahymena thermophila (Cate et al., 1996). Each nucleotide includes one base. There are four main types of bases, A, U, G, and C that may form pairs, with a strong preference for the pairs A-U and G-C. **(A)** a diagram representing the RNA as an open circle, with edges representing the base pairs. The presence of crossing edges indicates topological constraints in the RNA. **(B)** The shape of the RNA may be characterized as a union of balls, with one ball per atom, whose geometry can be characterized using the alpha shape theory (Edelsbrunner and Koehl, 2005). **(C)** The atomic displacements corresponding to the normal mode with lowest frequency are shown as (blue) line segments. Panel **(A)** was drawn using RNA 3D Hub (http://rna.bgsu.edu/rna3dhub), and panels **(B,C)** are drawn with Pymol (http://www.pymol.org).

Molecular structure, or shape is highly correlated to chemical reactivity as the latter depends on the positions of the nuclei and electrons within the molecule. Indeed, chemists have long used three-dimensional plastic and metal models to understand the many subtle effects of structure on reactivity and have invested in experimentally determining the structure of important molecules. The same applies to biochemistry, where structural genomics projects are based on the premise that the structure of biomolecules implies their function. As finding the high-resolution structure of a biomolecule by experimental methods remains a challenge, it is natural to turn to modeling. One would like to infer the geometry of a biomolecule from its primary sequence. This is the structure prediction challenge, nicknamed the “holy grail” of computational structural biology. A perhaps surprising finding from decades of research is that geometric reasoning plays a major role in attempts to solve this challenge, hinting at a more significant role for mathematics in this field. Figure 1 illustrates some possible contributions of geometry and topology for RNA structure prediction. Namely,

1. ***Topology and RNA structure (panel A).*** It is well established that topology plays an important role in defining the three dimensional conformations of RNA molecules (Bailor et al., 2010). To visualize RNA topology, a graph is constructed by representing the RNA backbone as a line and joining base pairs by edges. The arrangement of the base pairs determines the structure of this graph, which is related to “simple” secondary structures (helices, loops) and also to configurations known as pseudoknots (when two edges cross). While the latter are not common, they are functionally important. It is therefore important to address the problem of their prediction. The presence and number of pseudoknots are reflected in the *genus*, a numerical invariant associated to the RNA graph. This genus is related to, but not identical to, what graph theorists call the genus of a graph. It is straightforward to compute and implies topological constraints on the conformation of the RNA it represents (Bon et al., 2008; Bon and Orland, 2011; Reidys et al., 2011). Much remains to be done, however, on which invariants to use to quantify genus, and on how to include this information in structure prediction algorithms.
2. ***Geometry and RNA structure (panel B).*** A common model represents a biomolecule as a union of balls, in which each ball corresponds to an atom. Properties of the biomolecule are then expressed in terms of properties of the union (Edelsbrunner and Koehl, 2005). For example, the potential ligand sites are detected as cavities (Edelsbrunner et al., 1998) while the interaction of the biomolecule with its environment is quantified through the surface area and/or volume of the union of balls (Eisenberg and McLachlan, 1986; Ooi et al., 1987). Even with this simple representation, many mathematical challenges remain. How do we define a metric on the space of biomolecular structures? What are good (geometric) measures of similarity, or complementarity between two biomolecules? Answers to these questions will help to understand biomolecular interactions in normal cellular functions and in host-pathogen interactions, as well as support research on therapeutic drug developments.

## Grand challenge II: the mathematics of biomolecular dynamics

The functions of many biomolecules strongly correlate with conformational changes in their structure space, a process usually referred to as their activation. This process for example is very much at the core of enzymatic activity, as an enzyme and its substrate usually go through structural transitions that favor the chemical reaction (Henzler-Widman and Kern, 2007; Henzler-Widman et al., 2007). The structures of these transition states are of great interest, especially for drug design. Many enzyme inhibitors have been engineered to be transition state analogs, i.e., to resemble the transition state of the enzyme substrate; this design is only possible if the transition state of the enzyme itself is known. The transition state, however, is very short lived and its structure cannot be studied by standard experimental methods from structural biology. Computational “morphing” is then a valuable alternative, where the word morphing may relate to simple geometric morphing techniques or to more complex transformations that account for the physics of the system. Given two conformations for a bio-molecule, the problem is to find a plausible path along its energy surface, where plausible usually refers to a path with minimal frustration, also referred to as the Minimum Energy Path (MEP) (Weinan and Vanden-Eijnden, 2010). In principle, a brute force molecular dynamics (MD) simulation would solve the Minimum Energy Path problem (Hartmann et al., 2014), as it is designed to simulate the dynamics of the system with atomistic details. However, the timescales required for pushing a system over an energy barrier scale exponentially with the barrier height. As a result, traditional MD has difficulty surmounting even small barriers in times that are computationally accessible (Vendruscolo and Dobson, 2011). Recently, a technique for spatial discretization of the molecular structure space designed to help overcome such problems, the so-called Markov State Models (MSMs) (Chodera et al., 2007; Pan and Roux, 2008; Metzner et al., 2009; Noé et al., 2009) has attracted a lot of attention; this technique remains computational costly and currently limited to studying small molecular systems.

How can we generate alternate approaches to these MD techniques for morphing a bio-molecule from one state into another? How smooth is the morphing? If the actual morphing is not smooth, can we approximate it with a smooth diffeomorphic mapping? To solve these problems, we will need better methods for sampling the energy surface in the space of conformations for the molecule of interest, as well as better mathematical models of what defines a MEP, especially when the energy surface is rugged. This calls for collaborations between mathematicians and theoretical physicists.

I believe that in the next 10 years we will see more and more collaborations between mathematicians, physicists and biologists with hopes to decipher the roles of biomolecules in life. I foresee that new theories and new models will be created to better address the challenges mentioned above. This is truly an exciting time for biochemistry.
